# Genetic Polymorphisms of the Human Cytochrome P450 1A1* (CYP1A1) *and Cervical Cancer Susceptibility among Northeast Thai Women

**DOI:** 10.31557/APJCP.2020.21.1.243

**Published:** 2020

**Authors:** Mayuree Wongpratate, Wannapa Settheetham-Ishida, Sophida Phuthong, Sitakan Natphopsuk, Takafumi Ishida

**Affiliations:** 1 *Department of Physiology, Faculty of Medicine, Khon Kaen University, Khon Kaen, *; 2 *Chulabhorn International College of Medicine, Thammasat University, Thailand, *; 3 *Unit of Human Biology and Genetics, Department of Biological Sciences, Graduate School of Science, University of Tokyo, Tokyo, Japan. *

**Keywords:** Genetic polymorphisms, CYP1A1, cervical cancer, risk

## Abstract

**Background:**

CYP1A1 is an enzyme in phase I of the cytochrome P450 (CYP) superfamily, and plays a key role in detoxification of carcinogens. Host genetic predisposition in the *CYP1A1 *may be associated with an increased susceptibility to cervical cancer.The study aimed to evaluate four common polymorphisms of the *CYP1A1* and cervical cancer susceptibility among Northeast Thai women.

**Methods:**

A case-control study was conducted involving 204 patients with squamous cell cervical cancer (SCCA) and 204 age-matched healthy controls. DNA was extracted from peripheral blood leucocytes. *CYP1A1* m1, m3, and m4 genotypes were detected using PCR-RFLP, whereas the *CYP1A1* m2 genotype was investigated using real-time PCR. Haplotype analysis was performed using PHASE algorithm version 2.1.1.

**Results:**

*CYP1A1* m3 was monomorphic. Association between the common *CYP1A1* polymorphisms, m1 and m2, and cervical cancer risk was not observed (*p*>0.05), nor was any association found between the m1–m2–m4 haplotype and cervical cancer risk (*p*>0.05). Interestingly, the CA genotype of *CYP1A1 *m4 was observed in 30.88% of the cervical cancer patients but was absent in healthy controls.

**Conclusion:**

Our results demonstrated a possible involvement of the *CYP1A1* m4 polymorphism but no other common polymorphisms (viz., m1, m2, and m3) in the risk for cervical cancer.This finding may be useful when screening for risk of cervical cancer among Northeast Thai women.

## Introduction

Cytochrome P450 1A1 (CYP1A1), an important enzyme in phase I of cytochrome P450 superfamily (CYPs), plays a key role in the detoxification of several xenobiotics (e.g., 7-ethoxyresorufin, theophylline, caffeine, and 7-ethoxycoumarin) and endogenous substances (e.g., 17β-estradiol and estrone) (Bozina et al., 2009). In addition to these substances, CYP1A1 has potential for catalyzing environmental procarcinogens such as polycyclic aromatic hydrocarbons (PAHs), leading to generation of reactive metabolites that are highly reactive towards DNA (Androutsopoulos et al., 2009). Accumulating evidence shows that* CYP1A1* polymorphisms are associated with an increased susceptibility to cancer (Bartsch et al., 2000; Bozina et al., 2009; Androutsopoulos et al., 2009); due to alterations to mRNA expression and enzymatic activity (Cosma et al., 1993; Crofts et al., 1994, and Schwarz et al., 2001). These changes may influence the balance between electrophilic molecules and reactive metabolites, leading to DNA adduct formation and the initiation of carcinogenesis (Attia, 2010).

Comprising seven exons and six introns, the *CYP1A1* is located on 15q22-24 (Masson et al., 2005). It is generally expressed in extrahepatic tissues, especially epithelial tissues (Bozina et al., 2009; Santes-Palacios et al., 2016). Among *CYP1A1* variant alleles, common variants include four single nucleotide polymorphisms (SNPs): T3801C (m1), A2455G (m2), T3205C (m3), and C2453A (m4) (Bozina et al., 2009). Ordinary, m1 and m3 are located in the 3’ noncoding region, giving rise to a *Msp*I restriction site; whereas m2 and m4 are located in exon 7, leading to the amino acid transition of isoleucine to valine on codon 462 and threonine to asparagine on codon 461, respectively (Li et al., 2004). To date, genetic polymorphisms of human *CYP1A1* have been widely studied for the susceptibility to various cancers (e.g., cancer of lung, oral, larynx, breast, thyroid, prostate, renal, cervix uteri, gastric, and colon) (Li et al., 2004; Gajecka et al., 2005; Little et al., 2006; Siraj et al., 2008; Wright et al., 2010; Li et al., 2016; Balaji et al., 2012; Agudo et al., 2014; Meng et al., 2015). Notwithstanding, to our knowledge there are no recent reports of any association between the four polymorphic loci of the *CYP1A1* and cervical cancer susceptibility (Sugawara et al., 2003; Juárez-Cedillo et al., 2007; Gutman et al., 2009; Roszak et al., 2014; Abbas et al., 2014; Tan et al., 2016; Jain et al., 2017). Our aim was thus to evaluate these four polymorphic loci of *CYP1A1 *for the cervical cancer susceptibility among Northeast Thai women.

## Materials and Methods

*Subjects*

This was a case-control study, including 408 female volunteers between 26 and 81 years of age-conducted at Srinagarind Hospital and KhonKaen Hospital in KhonKaen Province, Northeast Thailand, between February 2009 and August 2011. The subjects included 204 cases of pathologically-defined squamous cell carcinoma of the cervix and 204 age-matched healthy women (5-year intervals) confirmed by cytological and histological examination.The sample size was obtained from the case-control sample size table where the level of significance, α = 0.05; the power = 0.9; β = 0.2; relative risk, R = 3.0; and proportion of mutant type, P_0_ = 0.05 (Schlesselman, 1982). Each subject was informed of the methodology and objectives of the research and signed an informed consent form. The study was reviewed and approved by the Ethics Committee of KhonKaen University (HE 571482).

*Detection of CYP1A1 polymorphisms*

Three milliliters of EDTA-blood samples were collected from all subjects. The genomic DNA was extracted from the buffy coat using GF-1 Blood DNA Extraction Kits (Vivantis, USA). Four polymorphisms (m1, m2, m3, and m4) of the *CYP1A1* were analyzed. The m1, m3, and m4 SNPs were examined using the polymerase chain reaction restriction fragment length polymorphism (PCR-RFLP) method (C1000TM Thermal Cycler 96W SYS, Bio-Rad, USA), while the m2 SNP was examined using TaqMan probe real-time PCR method (7500 Fast Real-Time PCR System, Applied Biosystems, USA). 

*The PCR condition and PCR reaction mixture are as follow*

*m2, m3, m4 SNP detection*– The method and sequence of primer pairs follow Hussein et al., (2014). The PCR reaction mixture contained 12.5 µL of 2X Taq Master Mix, 0.2 µL of each primer, 11.1 µL of distilled water, and 1 µL of genomic DNA (80 ng/µL), reaching a final volume of 25 µL. The following condition was used to amplify: a holding stage at 95°C for 9 minutes, followed by 35 cycles of denaturation at 94°C for 60 seconds, annealing at 57°C for 45 seconds, and extension at 72°C for 45 seconds, and the final elongation step at 72°C for 4 minutes. The PCR product was digested using the *Msp*I enzyme (New England, USA) in 1 UL of l0x NE buffer (New England. USA), incubated at 37°C for 2 hours confirmed by 2 % agarose gel electrophoresis, followed by ethidium bromide staining, and photographed under UV light. 

*m2 SNP detection*

The PCR reaction of 10 µL included 5 µL of TaqMan Universal PCR Master Mix, 0.5 µL of TaqMan probe with primers, 3.5 uL of distilled water, and 1 µL of genomic DNA. The holding stage was at 95°C for 10 minutes, followed by 40 cycles of denaturation at 95°C for 15 seconds, annealing at 60°C for 60 seconds, and extension at 60°C for 60 seconds. The TaqMan^®^ probe with primers (Applied Biosystems, USA) was designed for a specific SNP (viz., SNP assay: C_25624888_50). The respective FAM and VIC fluorescence dyes on the probes were used for the wild type (A allele) and the mutant type (G allele) (Balaji et al., 2012).

*m3 SNP detection*

The method and sequence of primer pairs followed Hirata et al., (2008). The PCR reaction mixture contained 12.5 µL of 2X Taq Master Mix, 0.2 µL of each primer, 9.1 µL of distilled water, and 3 µL of genomic DNA, reaching a final volume of 25 µL. The holding stage was at 94°C for 9 minutes, followed by 35 cycles of denaturation at 94°C for 30 seconds, annealing at 54°C for 40 seconds, and extension at 72°C for 40 seconds, with the final elongation step at 72°C for 4 minutes. The *Msp*I enzyme (New England, USA) was used to distinguish the m3 genotype, incubated at 37°C for 2 hours and confirmed by 2 % agarose gel electrophoresis with GelRed (DNA dye–ViSafe Red Gel Stain, Vivantis, USA).

*m4 SNP detection*

The method and sequence of primer pairs followed Cascorbi et al., (1996). The PCR reaction mixture contained 12.5 µL of 2X Taq Master Mix, 0.5 µL of each primer, 9.5 µL of distilled water, and 2 µL of genomic DNA, reaching a final volume of 25 µL. The holding stage was at 94°C for 5 minutes, followed by 35 cycles of denaturation at 94°C for 30 seconds, annealing at 61°C for 30 seconds, and extension at 72°C for 30 seconds, with a final elongation step at 72°C for 4 minutes. The *Bsa*I enzyme (New England, USA) was used to distinguish the m4 genotype, incubated at 37°C for 4 hours, and confirmed by 2.5% agarose gel electrophoresis with GelRed (DNA dye–ViSafe Red Gel Stain, Vivantis, USA).

*Statistical analyses*

Statistical analyses were performed using STATA software V.14. The association between each SNP of the *CYP1A1* polymorphisms and cervical cancer risk was assessed using uni- then multivariate logistic regression. The Hardy-Weinberg equilibrium was tested using Pearson’s chi-square. The haplotype analysis of the *CYP1A1* was performed using PHASE algorithm version 2.1.1. The strength of association between the *CYP1A1 *polymorphisms and cervical cancer risk was measured using odds ratios (ORs) with 95% conﬁdence intervals (CIs). A *p*-values of <0.05 was considered as statistically significant.

**Table 1:** *CYP1A1* m1, m2, m3 and m4 Polymorphisms and Cervical Cancer Risk

Polymorphisms	Genotypes (Allele)	Cases n (%)	Controls n (%)	Crude OR [95% CI, *p*]	Adjusted OR^a^ [95% CI, *p*]
m1 T3801C Intron	TT	46 (22.55)	52 (25.49)	1	1
	TC	104 (50.98)	100 (49.02)	1.18 [0.70-1.96, 0.5107]	1.51 [0.74-3.08, 0.262]
	CC	54 (26.47)	52 (25.49)	1.17 [0.65-2.11, 0.5676]	1.98 [0.87-4.48, 0.103]
	TC+CC	158 (77.45)	152 (74.51)	1.18 [0.73-1.90, 0.4869]	1.65 [0.84-3.24, 0.147]
	(T)	0.48	0.5	1	NA
	(C)	0.52	0.5	1.084 [0.82-1.43, 0.5695]	NA
m2 A2455G *Ile462Val* Exon 7	AA	96 (47.06)	99 (48.53)	1	1
	AG	90 (44.12)	90 (44.12)	1.03 [0.67-1.58, 0.8817]	1.04 [0.57-1.80, 0.899]
	GG	18 (8.82)	15 (7.35)	1.24 [0.55-2.80, 0.5723]	2.54 [0.88-7.35, 0.085]
	AG+GG	108 (52.94)	105 (51.47)	1.06 [0.70-1.59, 0.7662]	1.19 [0.67-2.09, 0.550]
	(A)	0.691	0.706	1	NA
	(G)	0.309	0.294	1.074 [0.79-1.46, 0.6427]	NA
m3, T3205C, Intron	TT	204 (100)	204 (100)	1	1
m4 C2453A *Thr461Asp* Exon7	CC	141 (69.12)	204 (100)	1	1
	CA	63 (30.88)	0 (0.00)	_b	_b
	AA	0 (0.00)	0 (0.00)	_b	_b
	(C)	0.85	1	1	NA
	(A)	0.15	0	_b	NA

NA, not applicable; OR, odds ratio; CI, confidence interval; ^a^adjusted multiple logistic regression for contraceptive use, partner smokes, and HPV infection; ^b^, drop because of a zero cell count

**Figure 1:**
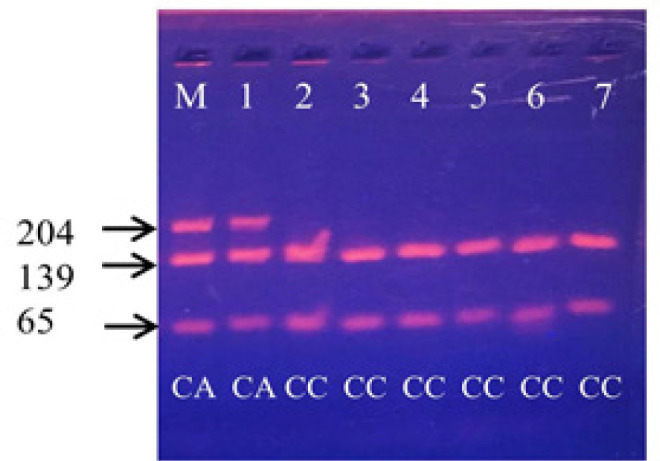
Example of Restriction Fragment Length Polymorphism (RFLP) of *CYP1A1* m4 Products: *Bsa*I enzyme was used to digest the 204-bp product of *CYP1A1 *m4, M is a molecular-weight size marker, the homozygous wild-type (CC) produced two bands, a 139-bp and 65-bp as shown in lanes 2, 3, 4, 5, 6 and 7, the heterozygous type (CA) produced three bands, a 204-bp, 139-bp and 65-bp as shown in lanes M and 1 and the homozygous mutant-type (AA) produced entirely 204-bp which was not detected in this study

**Table 2:** *CYP1A1* m1-m2-m4 Haplotype and Cervical Cancer Risk

*CYP1A1 *haplotypes (m1-m2-m4)	Cases n (%)	Controls n (%)	OR[95% CI, *p*], T-A-C as reference
T-A-C	163 (39.95)	200 (49.02)	1
T-A-A	33 (8.09)	0 (0.00)	_b
T-G-C	0 (0.00)	4 (0.98)	_b
T-G-A	0 (0.00)	0 (0.00)	_b
C-A-C	75 (18.38)	88 (21.57)	1.046 [0.72-1.52, 0.8132]
C-A-A	11 (2.70)	0 (0.00)	_b
C-G-C	106 (25.98)	116 (28.43)	1.121 [0.80-1.57, 0.5030]
C-G-A	20 (4.90)	0 (0.00)	_b

OR, odds ratio; CI, confidence interval; ^b^drop because of a zero cell count

## Results

Genotype distributions and allele frequencies of the four loci of the *CYP1A1* are summarized in Table 1. One locus, *CYP1A1* m3, was monomorphic. The A allele of m4 was detected only in the cases (CA=63/204 cases, 30.88%) but the AA genotype was not detected (Figure 1). No association was found between the m1, m2, and m4 genotypes and cervical cancer risk (*p*>0.05). The genotype distributions of the m1 and m2 polymorphisms among the controls and cases were consistent with the Hardy-Weinberg equilibrium (HWE) (*p*>0.05); however, that of the m4 polymorphism among the cases deviated from the HWE (*p*<0.01). The presence of the A allele carrying chromosomes being only among the cases was an unexpected finding (Fisher’s exact test *p*<0.0001). We then evaluated the characteristics of the m4 A allele carriers. Among the 204 cases, the A allele carriers did not show (a) age dependency, (b) different HPV positivity, or (c) difference in age of menarche.

The haplotype analysis of *CYP 1A1* m1–m2–m4 is shown in Table 2. Taking T–A–C as the reference, a relationship between the m1–m2–m4 haplotype and cervical cancer risk was not observed (*p*>0.05).

## Discussion

We evaluated the association between four common polymorphisms in the human *CYP1A1* and development of cervical cancer among Northeastern Thai women. In this study, the m3 locus was monomorphic as in other reports for Indians (Singh et al., 2007) and Americans (San Francisco) (Hirata et al., 2008). The polymorphic status in the m3 locus may be specific for persons of African-American descent (Garte et al., 2001; Li et al., 2004). Our results show a lack of significant association between m1 and m2 polymorphisms and cervical cancer risk, which as in several others tudies-done among Japanese, Israeli Jewish, Polish, Chinese, and Indian populations (Sugawara et al., 2003; Gutman et al., 2009; Roszak et al., 2014; Tan et al., 2016). To contrast, significant associations between m1 and/or m2 polymorphism and increased cervical cancer risk have been documented in several populations (Tan et al., 2017; Juárez-Cedillo et al., 2007; Jain et al., 2017; Li et al., 2016; Wang et al., 2017; Ding et al., 2018), and a meta-analysis indicated that the m1 (CC) genotype was associated with an increased risk for cervical cancer among Asians and Mixed populations (Wu et al., 2013). It is thus premature to conclude the role of m1 and m2 polymorphisms in cervical cancer development.

No significant association was found between the m1–m2–m4 haplotypes and cervical cancer risk in the current study (*p*>0.05). At present, only a few studies have reported an association between *CYP1A1 *haplotypes and cancer susceptibility. Wright et al.,(2010) and Shah et al., (2008) showed that when using the T–A–C haplotype as the reference, the C–G–C and T–G–C haplotypes were associated with an increased risk of lung cancer. To compare, Chang et al., (2003) showed that the C–A–C haplotype was associated with a decreased risk of prostate cancer via the role of estrogen metabolism. These inconsistent findings may indicate a specific role of the *CYP1A1* haplotypes in different types of cancer.

As for m4 polymorphism, possible associations have been documented between the A allele and risk for lung cancer (Gallegos-Arreola et al., 2008; Shah et al., 2008; Ezzeldin et al., 2017), laryngeal squamous cell carcinoma (Gajecka et al., 2005) and thyroid cancer (Siraj et al., 2008). No association has been observed between m4 polymorphism and breast, colorectal, and gastric cancer (Li et al., 2004; Singh et al., 2007; Amrani et al., 2016, Little et al., 2006; Agudo et al., 2014). Moreover, none of the previous studies have documented any association between m4 polymorphisms and cervical cancer; notwithstanding, our finding that the A allele existed only among cases (*p*<0.0001) strongly suggests participation of the *CYP1A1* m4 A allele in the development of cervical cancer. We attempted to determine the cause of the high concentration of the A allele among cases (at least 60-fold over that of the controls, data not shown); by analyzing personal parameters.We then considered with respect to CYP1A1 function in the metabolic activation of carcinogens. 

Benzo[a]pyrene-an environmental procarcinogen-is a specific substrate for CYP1A1 activation leading to generation of highly reactive intermediates-such as B[a]P-7,8-diol-9,10-epoxide (BPDE), which is classified as the potent carcinogenic metabolite (Bozina et al., 2009; Androutsopoulos et al., 2009). BPDE-DNA adducts have been identified in human epithelial cervical tissue, especially among smokers (Melikian et al., 1999). In human tissues,* CYP1A1* mRNA expression in the cervix is low (Hu et al., 2016); however, high up-regulation of the *CYP1A1* mRNA, protein, and activity can be detected by inducers (e.g., polycyclic aromatic hydrocarbons) (Nebert et al., 2004). While CYP1A1 activity trends to be highest for the m4 variant in benzo[a]pyrene metabolism (Schwarz et al., 2001), the m4 variant exhibits the greatest catalytic efficiency for producing diol species and significantly increases formation of diol epoxide-2 (BPDE2)—the potent mutagenic species associated with increased cancer risk (Schwarz et al., 2001; Rubin, 2001). Smoking is a major risk factor for development of cervical cancer among Northeast Thai women (Natphopsuk et al., 2012). Although our smokers were passive smokers, those with the A allele may be more vulnerable to the carcinogenicity of smoke than those with the CC genotype. There is thus a possibility that the m4 variant of the A allele plays a key role in the development of cervical cancer.

In summary, our results demonstrated the possible involvement of the *CYP1A1* m4 polymorphism over against other common polymorphisms (m1, m2, and m3) in the risk for cervical cancer among Northeast Thai women.
